# Comparative analysis of the sensitivity and specificity of the classification criteria and correlation with prognosis of disease in patients with Systemic Lupus Erythematosus

**DOI:** 10.31138/mjr.29.4.232

**Published:** 2018-12-18

**Authors:** Christina Adamichou, Dionysios Nikolopoulos, Emmanouil Papastefanakis, Eleni Kalogiannaki, Irini Gergianaki, Aggeliki Kountouri, Argyro Repa, Nestor Avgoustidis, Nikolaos Kougkas, Prodromos Sidiropoulos, Antonis Fanouriakis, George Bertsias

**Affiliations:** 1 Department of Rheumatology, Clinical Immunology and Allergy, University of Crete School of Medicine, Heraklion, Greece,; 2 Rheumatology Clinic, ‘Attikon’ University Hospital, Athens, Greece

**Keywords:** Systemic Lupus Erythematosus, classification criteria

## Abstract

**Background:**

Systemic lupus erythematosus (SLE) is a chronic autoimmune disease characterized by significant clinical heterogeneity with early diagnosis being a major challenge, complicated by the absence of formal diagnostic criteria. Instead, classification criteria have been developed to enable the homogenous inclusion of patients in clinical trials, with the most commonly used those of the American College of Rheumatology (ACR 1997) and the Systemic Lupus International Collaborating Clinics Classification Criteria (SLICC 2012). These criteria are widely used in clinical practice as diagnostic tools, although they fail to diagnose up to 20% of patients with SLE or may delay diagnosis. These restrictions have led to the recent (2018) introduction of new classification criteria jointly by the European League Against Rheumatism (EULAR) and ACR.

**Aims of the Study:**

We will compare the sensitivity and specificity of the earlier and new classification criteria after a systematic analysis (retrospective study) of a group of SLE patients. In addition, we will examine which set of criteria permits the earliest classification of the disease in a prospective cohort of patients with undifferentiated connective tissue disease (UCTD). The prognostic impact (permanent organ damage) of the classification of SLE patients with the three sets of criteria will also be examined.

**Methods:**

Data from the existing Cretan lupus registry will be used to retrospectively include consecutively registered patients aged ≥15 years diagnosed with SLE during 01/2005–12/2016 by an expert physician and followed-up for at least 6 months. All sets of criteria (ACR 1997, SLICC 2012, EULAR/ACR 2018) will be tested at the time of physician-based diagnosis and also at last follow-up. A prospective study arm will include cases with a diagnosis of UCTD and will be followed-up in the outpatient clinic for 3–5 years.

**Anticipated Benefits:**

This is the first study to include the application of the new criteria (EULAR/ACR 2018) to a group of SLE patients. Determining their diagnostic value in comparison to existing criteria or diagnosis by a specialist will provide important information both for the value of their application at the level of clinical studies and for their use in clinical practice as diagnostic criteria.

## INTRODUCTION

### Background/rationale

Systemic lupus erythematosus (SLE) is a chronic autoimmune disorder that can present with a broad spectrum of manifestations from different organs, which can be of variable specificity and severity. As a consequence, disease diagnosis can be challenging; especially at the early stages, which is further complicated by the absence of specific diagnostic criteria. Instead, classification criteria have been developed, which are based on a combination of clinical and laboratory / immunological findings. The most commonly and widely used are those of the American College of Rheumatology (ACR 1997).^1^ Despite their ease of use, the ACR 1997 criteria have low sensitivity for severe forms of the disease, and can classify as SLE patients with pure mild mucocutaneous manifestations. In addition, they do not encompass several manifestations from some organs / systems (e.g. nervous, hematopoietic); possibly resulting in delayed diagnosis. In 2012, the Systemic Lupus International Collaborating Clinics (SLICC) proposed new classification criteria, which are advantageous in including additional number of clinical (e.g. neurological) and immunological (e.g. antiphospholipid antibodies, low C3 / C4) features.^2^ Despite their higher sensitivity (92–97%), the SLICC 2012 criteria lack specificity (74–88%).^2–5^ These sets of criteria (ACR 1997, SLICC 2012) were primarily developed to select homogeneous patient populations in epidemiological or clinical studies. However, they are widely used in clinical practice for diagnostic purposes,^6^ with significant limitations, as they can miss a number of SLE patients or result in diagnostic delays.^7^ The development of optimized classification (or diagnosis) criteria is an important and unfulfilled goal. These concerns have led to the recent (2018) cooperation of EULAR/ACR for the introduction of a new set of classification criteria. The new criteria require the existence of a positive antinuclear antibody (ANA) title as an entry criterion, coupled with a list of variably-weighed clinical and immunological criteria for the calculation of the total score that will classify or not the patient as SLE.^8^

To date, there have been no studies validating the sensitivity and specificity of the new EULAR/ACR criteria as compared to the previous criteria, and it is currently unknown whether the new EULAR/ACR criteria have indeed increased diagnostic accuracy. Importantly, the prognostic implications of classifying SLE patients with either of the three sets of classification criteria is not known. Furthermore, existing published data on the diagnostic value of the classification criteria have mainly been derived from selected SLE patients monitored in tertiary centres and therefore cannot be generalized in the community.

## METHODS

### Setting and participants

This is a non-invasive observational study, with a retrospective and a prospective arm. During the *first phase*, data from the existing SLE patient registry that have been developed since 2012 at the Rheumatologic Department of the University Hospital of Heraklion (PAGNI)^9^ will be exploited. The registry contains demographic and clinical data from medical records of patients entering a secure, specially configured electronic database that is installed in the Rheumatology Department on the protected server and network of PAGNI. The operation and maintenance of the database is strictly supervised by the scientifically accountable protocol and access is granted only to authorized users / researchers. All principles of anonymity, confidentiality and non-traceability of data are adhered to.

Cases diagnosed as SLE according to expert physician judgment during the period 01/2005–12/2016 will be identified and all necessary data and variables will be recorded. For a patient to be eligible for inclusion in the study, sufficient documentation data should be available, i.e. name, surname and at least two of three identifiers (date of birth, father’s first name and Social Security Number). A follow-up of at least 6 months since SLE diagnosis is required for inclusion and a known ANA status. Patients diagnosed before the age of 15, those with a diagnosis before the year 2005 or after 12/2016, patients with cutaneous lupus and drug-induced lupus will be excluded. Furthermore, patients with a follow-up of less than 6 months after the diagnosis of SLE will not be included. Patients with incomplete/insufficient data and damaged folders will also be excluded.

During the *second phase*, patients diagnosed as ‘’possible SLE”, incomplete lupus or UCTD will be identified and will be a separate group of patients monitored in the out-patient clinic of the Rheumatology Department of Heraklion every 6–12 months for 3–5 years. During each visit, the presence of the items of each classification criteria will be recorded and the opinion of a specialist whether a diagnosis of SLE exists or not. These groups of patients are known to diagnose with SLE at a frequency of 20–40% after 2–5 years.^10,11^ The aim of the prospective phase will be to assess if and which of the three classification criteria allows the earlier classification / diagnosis of SLE patients.

Finally, a number of patients with other rheumatological diagnosis will be grouped as disease controls. They will be randomly selected from the existing registry of patients with rheumatological diseases of the Rheumatology Department of Heraklion and the same data will be documented from the patient folders.

### Variables

From each SLE patient chart, data will be collected retrospectively, which include: demographics (gender, ethnicity, date of birth), date of SLE diagnosis (according to the physician), presence (yes/no) and year of appearance of each of the classification criteria items, presence (yes/no) and year of appearance of selected additional items (including Raynaud’s, lymphadenopathy-splenomegaly, sicca, SSA/SSB, etc.), date of last follow-up visit/assessment, organ damage (SLICC/ACR Damage Index)^12^ and severity of SLE. SLE is characterized as mild, moderate or severe, based on the presence of British Isles Lupus Assessment Group (BILAG) severity of manifestations,^13^ the use of immunosuppressants, and physician’s global assessment. The same variables will be collected prospectively for the UCTD cases that will be followed-up for 2–5 years.

### Data collection

For the collection and management of both retrospective and prospective study databases, the RedCap online platform is used. On this platform, the data is recorded in six different fields for each patient including:

1. Demographics (patient number, gender, nationality, date of birth, date of SLE diagnosis and date of last visit);
2. ACR criteria (11 items and date of appearance of each individual item);
3. SLICC criteria (18 items and date of appearance of each individual element);
4. New criteria and elements that do not fit into a set of criteria;
  - EULAR/ACR 2018 criteria (20 items and date of appearance of each individual element), and
  - Non-included criteria (24 items and date of each individual item);
5. Severity index (22 items and year of each component); and
6. SLICC damage index (42 items and year of appearance).

For the prospective data collection, each patient will have the 6 fields filled in at baseline and at each follow-up visit (for fields 2–6). Furthermore, the patient’s diagnosis will be reviewed during every visit, as to whether this is still a UCTD case, or whether this has changed diagnosis to SLE or any other autoimmune disease. A similar instrument will be used for the control group, that will include only the first 4 fields.

## AIMS OF THE STUDY

This observational study aims to compare the sensitivity and specificity of existing sets of SLE classification criteria (ACR 1987, SLICC 2012, EULAR/ACR 2018) against physician-based diagnosis, which will be our study’s gold standard for SLE diagnosis. Secondary endpoints will be to determine which – if any – of the classification criteria allow for the earlier classification/diagnosis of SLE patients, to test whether specific additions (e.g. Raynaud’s), amendments or combinations in the existing classification criteria can enhance their sensitivity in diagnosing SLE and to examine the outcome of SLE patients who are classified exclusively by either of the classification criteria with regards to organ damage accrual and disease severity.

## ANTICIPATED BENEFITS

This will be the first study to include the application of the new criteria (EULAR/ACR 2018) to a group of SLE patients, as well as their comparison with the previous set of criteria. Determining the sensitivity and specificity of the new criteria for the classification / diagnosis of SLE patients will be important information both for the value of their application at the level of clinical studies and for their use in clinical practice as diagnostic criteria. As is well known, in order to use a set of criteria as a diagnostic in a disease with so much heterogeneity of manifestations and gravity, both its sensitivity and its specificity should approach 100%. Therefore, applying the new criteria to a well-defined patient cohort will bring interesting results for their future use in clinical practice.

## STUDY APPROVAL

The study has been approved by the Ethics Committee of the University Hospital of Heraklion, Crete, Greece.
